# Epigenome-wide association study of attention-deficit/hyperactivity disorder in adults

**DOI:** 10.1038/s41398-020-0860-4

**Published:** 2020-06-19

**Authors:** Paula Rovira, Cristina Sánchez-Mora, Mireia Pagerols, Vanesa Richarte, Montserrat Corrales, Christian Fadeuilhe, Laura Vilar-Ribó, Lorena Arribas, Gemma Shireby, Eilis Hannon, Jonathan Mill, Miquel Casas, Josep Antoni Ramos-Quiroga, María Soler Artigas, Marta Ribasés

**Affiliations:** 1 Psychiatric Genetics Unit, Group of Psychiatry, Mental Health and Addiction, Vall d’Hebron Research Institute (VHIR), Universitat Autònoma de Barcelona, Barcelona, Spain; 2 Department of Psychiatry, Hospital Universitari Vall d’Hebron, Barcelona, Spain; 3 Biomedical Network Research Centre on Mental Health (CIBERSAM), Instituto de Salud Carlos III, Madrid, Spain; 4 Department of Genetics, Microbiology, and Statistics, Faculty of Biology, University of Barcelona, Catalonia, Spain; 5 Department of Psychiatry and Legal Medicine, Universitat Autònoma de Barcelona, Barcelona, Spain; 6 University of Exeter Medical School, University of Exeter, Exeter, UK

**Keywords:** ADHD, Genetics

## Abstract

Attention-deficit/hyperactivity disorder (ADHD) is a highly heritable neurodevelopmental disorder that often persists into adulthood. There is growing evidence that epigenetic dysregulation participates in ADHD. Given that only a limited number of epigenome-wide association studies (EWASs) of ADHD have been conducted so far and they have mainly focused on pediatric and population-based samples, we performed an EWAS in a clinical sample of adults with ADHD. We report one CpG site and four regions differentially methylated between patients and controls, which are located in or near genes previously involved in autoimmune diseases, cancer or neuroticism. Our sensitivity analyses indicate that smoking status is not responsible for these results and that polygenic risk burden for ADHD does not greatly impact the signatures identified. Additionally, we show an overlap of our EWAS findings with genetic signatures previously described for ADHD and with epigenetic signatures for smoking behavior and maternal smoking. These findings support a role of DNA methylation in ADHD and emphasize the need for additional efforts in larger samples to clarify the role of epigenetic mechanisms on ADHD across the lifespan.

## Introduction

Attention-deficit/hyperactivity disorder (ADHD) is a common neurodevelopmental disorder characterized by age-inappropriate levels of inattention, impulsivity and hyperactivity^1^. ADHD is a disabling condition in childhood and adolescence which often persists into adulthood, interfering with the quality of social, academic, or occupational functioning^2,3^.

ADHD is a multifactorial disorder with an estimated heritability of 76%. Twenty-two percent of its phenotypic variance is explained by common genetic variants^1,4^ and the proportion of variance still to be explained might be, to some extent, accounted for by gene by environment interactions. In this context, epigenetic processes have emerged as a plausible mechanism by which environmental exposures can lead to long-lasting alterations, such as variation in brain structure or neuronal circuits, found in psychiatric disorders^5–7^. There is growing evidence that epigenetic dysregulation is a feature of ADHD^6,8–11^, depression^12^, autism^13–16^, schizophrenia^17,18^ and bipolar disorder^19^.

Studies of DNA methylation profiles in ADHD have been conducted using peripheral blood, cord blood, buccal samples or saliva^6,9–11,20–28^. Candidate gene studies have revealed differential methylation patterns in genes involved in the dopaminergic, serotoninergic and neurotrophic systems, including *SLC6A4*, *DRD4, COMT, ANKK1, BDNF*, or *NGFR*, associated with ADHD symptomatology and severity^23–28^. Seven epigenome-wide association studies (EWASs) on ADHD have been run to date, with sample sizes ranging from 54 subjects for clinical samples^21^ to 4,689 individuals in a meta-analysis considering ADHD symptomatology in general population^9^, yielding non-overlapping findings across them^6,9–11,20–22^. There is limited research on adults using this approach, given that most of the EWASs have focused on pediatric samples^6,10,11,20,22^. To the best of our knowledge, only two studies evaluated methylome-wide patterns on adults^9,21^. One identified methylation changes associated with ADHD symptomatology that did not remain significant when results were meta-analyzed across cohorts^9^. The second one found hypermethylated regions in genes involved in fatty acid metabolism and fatty acid oxidation pathways associated with ADHD persistence when compared to remittance^21^. In the childhood period, Wilmot et al. analyzed a population cohort of school-age boys and found lower methylation levels at the *VIPR2* gene in ADHD subjects compared to their age- and sex- matched controls^10^, results that were recently replicated in the largest EWAS on ADHD in children conducted so far^22^. In a similar aged population cohort, Walton et al. investigated ADHD symptom trajectories from birth to adolescence and pointed to epigenetic marks in genes related to neural tube development and peroxisomal mechanisms as candidates to be involved in the different ADHD symptom trajectories across time^6^. In the most recent EWAS evaluating ADHD symptoms in population-based cohorts, aberrant methylation patterns at birth in different regions, lying in the *ERC2* and *CREB5* genes among others, were associated with later ADHD symptoms in childhood or adolescence^11^. And finally, the latest and largest EWAS conducted in a clinical sample of children with ADHD supported the association between ADHD polygenic risk and DNA methylation patterns at the *GART* and *SON* genes^22^.

Recent evidence supports a large genetic overlap between ADHD in children and adults^29^, but little is known about the co-occurrence between the epigenetic signatures characterizing both groups of age. In addition, although various studies report shared genetics between ADHD and several psychiatric and behavioral traits^4,29^, this overlap has not been assessed yet using epigenome-wide data.

Whereas most previous studies considered pediatric clinical samples or adult population-based cohorts with measures of ADHD symptoms, we report an EWAS on a clinical sample of adults with ADHD. With these data we (i) assessed DNA methylation signatures for ADHD in adults through an EWAS in peripheral blood mononuclear cells, (ii) tested whether either polygenic risk burden for ADHD or smoking status had an impact on those DNA methylation signatures, (iii) examined whether exposure to stressful life events had an effect on these methylation patterns in ADHD subjects and (iv) explored the overlap between these findings and results from previous meta-analyses of genome-wide association studies (GWAS-MA) on clinical ADHD or ADHD symptoms in population-based samples, and EWAS on ADHD symptoms or exposure to stressful life events.

## Materials and methods

### Participants and clinical assessment

The clinical sample consisted of 103 ADHD subjects that were referred to an ADHD program from primary care centers and adult community mental health services. All subjects were evaluated and recruited prospectively from a restricted geographic area of Catalonia (Spain) in a specialized out-patient program for Adult ADHD and by a single clinical group at Hospital Universitari Vall d’Hebron of Barcelona (Spain).

The clinical assessment consisted of structured interviews and self-reported questionnaires in two different steps: (i) assessment of ADHD diagnosis based on symptomatology using the Conner’s Adult ADHD Diagnostic Interview for DSM-IV (CAADID) by a psychiatrist and, (ii) assessment of the severity of ADHD symptoms, the levels of impairment and the presence of comorbid disorders by a psychologist to increase the diagnostic accuracy and reduce the likelihood of misdiagnosis with the Conners ADHD Rating Scale (CAARS), the ADHD Rating Scale (ADHD-RS), the Clinical Global Impression (CGI), the Wender Utah Rating Scale (WURS), the Sheehan Disability Inventory (SDS), and the Structured Clinical Interview for DSM-IV Axis I and II Disorders (SCID-I and SCID-II). Afterwards, the psychiatrist and psychologist integrate the clinical information and self-reports for the valid assessment of symptoms and impairments. In case of discordance between different raters of ADHD symptoms or inconsistencies between reporters in responses to items measuring similar symptoms, the clinician-identified symptoms on the CAADID prevailed. Exclusion criteria were IQ < 70; lifelong and current history of mood, psychotic, anxiety, substance abuse, and DSM-IV axis II disorders; pervasive developmental disorders; a history or the current presence of a condition or illness, including neurologic, metabolic, cardiac, liver, kidney, or respiratory disease; a chronic medication of any kind; birth weight≤1.5 kg; and other neurological or systemic disorders that might explain ADHD symptoms. For more detailed information on clinical assessment see Sánchez-Mora et al.^30^.

Data pertaining to exposure to 17 stressful life events (six gestational and 11 postnatal) were collected retrospectively with the CAADID Part I^31^ and were available from 98 subjects with ADHD. No information was available from controls. Specifically, this questionnaire includes: premature birth, illegal drug abuse during pregnancy, maternal smoking, prenatal exposure to drugs, maternal health problems during pregnancy, other problems during maternal pregnancy, exposure to heavy metals, malnutrition, financial stress and/or poverty, extreme familial stress, neglect, familiar violence, emotional and physical maltreatment, sexual abuse, death or separation from a loved one, and other trauma in childhood or adolescence.

The control sample consisted of 100 unrelated healthy blood donors matched by sex and ethnicity with the clinical group. Individuals with ADHD symptomatology were excluded retrospectively under the following criteria: (1) having been diagnosed with ADHD previously or (2) answering positively to the lifetime presence of the following ADHD symptoms: (a) often has trouble in keeping attention on tasks, (b) usually loses things needed for tasks, (c) often fidgets with hands or feet or squirms in seat, and (d) often gets up from seat when remaining in seat is expected.

All subjects reported European ancestry, which was confirmed through principal component analysis (PCA) using genetic data. The study was approved by the Clinical Research Ethics Committee (CREC) of Hospital Universitari Vall d’Hebron, all methods were performed in accordance to the relevant guidelines and regulations and written informed consent was obtained from all subjects before inclusion into the study.

### DNA isolation, quantification, and genome-wide DNA methylation assays

Peripheral blood mononuclear cells (PBMCs) of patients with ADHD and controls were isolated using the Ficoll density gradient method, and DNA was extracted using the QIAamp DNA Mini Kit DNA Purification following manufacturer’s instructions (Qiagen, Hilden, Germany). The quality of the samples was checked by NanoDrop^®^ ND-1000 (Thermo Fisher Scientific, MA) and by PicoGreen^®^ (Thermo Fisher Scientific, MA). Genome-wide DNA methylation was assessed with the Illumina Infinium MethylationEPIC BeadChip Kit (EPIC array) (Illumina, San Diego, CA, USA) following sodium bisulfite treatment of genomic DNA.

### DNA methylation analysis based on ADHD diagnosis

#### Data preprocessing and normalization

The 203 samples included in this study were assayed in three batches, which were preprocessed and normalized separately. Raw signal intensities of each probe were extracted using the Illumina Genome Studio software (https://support.illumina.com) and were imported into the R software (3.6.0 version; https://www.R-project.org) using the minfiData 0.2 package^32^. The bisulfite conversion control probes and the 59 single nucleotide polymorphism (SNP) probes of the EPIC array were used to calculate the bisulfite conversion reaction efficiency and to confirm the absence of sample contamination, respectively. Sex was confirmed for all samples using the *getSex* function of the minfi R package^33^. The Horvath Epigenetic Clock algorithm^34^ implemented by the *agep* function of the wateRmelon R package was used to calculate the estimated age of participants according to their DNA methylation data, which correlated with their reported age (ρ = 0.82, SE = 0.04, *P* < 2.00E−16). Poorly performing probes or samples were removed using the wateRmelon R package (version 0.9.9;^35^). The exclusion criteria for the probes included detection *P*-values >0.05 for >1% of the samples and a beadcount <3 for >5% of the samples. Probes that were cross-reactive, present in sexual chromosomes or that contained polymorphisms were also excluded from the study^36,37^. Samples with >1% of probes with a detection *P*-value >0.01 were also removed. Probes that passed the quality control filters were quantile normalized with the *dasen* function of the wateRmelon R package.

#### Bioinformatic and statistical analyses

PCA of methylation values was conducted using the *prcomp* function of the stats R package, first separately for each batch and then across all batches. Within batch, non-biological experimental variation (Sentrix Position and chip ID) of normalized methylation values was tested for association with the Principal Component loadings (PCs). Chip ID was associated with the first PC (PC1) in all three batches, which accounted for the 99% of the variation of samples. We therefore adjusted the beta values with the *ComBat* function of the SVA R package^38^ for this variable. The effect of batch and sex on adjusted methylation values of probes present in the three batches after quality control (*n* = 744,227) was tested for association with the PCs estimated in the overall sample. Evidence of clustering according to batch was visually detected and statistically confirmed with a significant association of PC1 with batch (*P*-value < 2.20E−16).

Given that detailed smoking information was not available for each individual, an individual smoking score (continuous measure) was generated based on DNA methylation sites known to be associated with current smoking using a method developed by Elliot and colleagues^39^. To account for methylation differences between cell types, we estimated the cell-type composition using the *estimateCellCounts* function of the FlowSorted.Blood.450k R package^40^.

Probe-wise differential methylation analysis was performed using the *lmFit* function of the limma R package^41^. Each CpG site was tested individually in a linear regression model with normalized, corrected beta values as the dependent variable and ADHD status as independent predictor, including covariates for sex, age, batch, smoking score and cell-type composition. Age was included as covariate in all the analysis, since it was significantly different between cases and controls. Multiple testing corrections were applied using false discovery rate (FDR) with a cut-off of 5%^42^. The qqman R package was used to generate the Manhattan plot.

The post-hoc power analysis in our sample calculated with the EPIC array online tool (https://epigenetics.essex.ac.uk/shiny/EPICDNAmPowerCalcs/)^43^ using the default significance threshold (*P*-value < 9.42E−08) showed that 6.12% of sites had > 90% power to detect a mean methylation difference of 1%.

At the differentially methylated CpG site, we tested the association between DNA methylation and the exposure to at least one stressful life event, and to each stressful life event separately using the *lmFit* function of the limma R package. As 17 stressful life events were tested, Bonferroni correction was set at *P* < 2.94E−03. We also tested the correlation between the number of stressful life events (sum of overall stressful life events and also separated in pre- and post-natal periods) and DNA methylation levels using Spearman’s correlation.

To identify differentially methylated regions (DMRs), we used the Python module *comb-p*^44^ to group spatially correlated CpG sites with a seed of *P*-value < 0.01 and 500 base pairs (bp) as the maximum distance. DMR *P*-values were corrected for multiple testing using the Šidák correction^45^ and significant regions were defined as those with at least two probes and an adjusted *P*-value < 0.05. DMRs were mapped to genes using the interface provided by the minfi R package or the UCSC Genome Browser to identify the closest gene when no genes were mapped to a region (https://genome.ucsc.edu/cgi-bin/hgGateway).

Sensitivity analyses were conducted with the same parameters described above for the probe-wise and regional analyses excluding smoking score as covariate in the model.

### DNA methylation analysis based on ADHD diagnosis controlling for ADHD polygenic burden

#### Bioinformatic and statistical analyses

ADHD polygenic burden was inferred using a Polygenic Risk Score (PRS) built in a subset of 195 individuals with genotype data available, from three different genotyping waves (Illumina HumanOmni1-Quad BeadChip (*n* = 3), Illumina HumanOmni2.5-8 BeadChip (*n* = 29) and Infinium™ Global Screening Array-24 v2.0 (*n* = 163) (Illumina, San Diego, CA, USA), using summary statistics of the largest GWAS-MA performed to date on ADHD^4^, with different *P*-value thresholds ((*P*_T_) < 1e−04, 5e−04, 0.001, 0.005, 0.01, 0.05, 0.1, 0.2, 0.3, 0.4, 0.5, 1). None of the samples used in this study were included in this GWAS-MA^4^, and thus did not contribute to defining the variants included in the PRS.

In this subset of 195 individuals, sensitivity analyses for the differentially methylated sites and regions were conducted with the same parameters used in the original EWAS but including the PRS explaining the most variance (Nagelkerke’s *R*^2^) as an additional covariate to control for ADHD polygenic risk burden.

ADHD PRSs for each individual were generated with PRSice2 (https://choishingwan.github.io/PRSice/) including sex and the first five PCs as covariates in the model. To set an empirical threshold for the best-fit PRS, 1,000 permutations were run. Information about the pre-imputation quality control at individual and SNP level for the 195 individuals in the target sample and about the phasing and imputation software used is described elsewhere^29^. The European ancestry panel of the 1000 Genomes Project was considered as reference for the imputation (ftp://ftp.1000genomes.ebi.ac.uk/vol1/ftp/) and best guess genotypes were filtered by excluding variants with MAF < 0.05, missing rate>0.01, Hardy-Weinberg Equilibrium (*P* < 1.00E−06). Ambiguous strand and multiallelic variants were removed and independent SNPs (obtained using the clumping parameters *p1* = 1, *p2* = 1, *r2* = 0.2, kb=250 in PLINK1.9^46^) present in all individuals were included (*n* = 37,527).

### Enrichment analyses

We assessed whether probes in different categories: (i) showing a statistically significant proportion of methylation variance explained by additive genetic effects as reported by Zeng et al.^47^; (ii) probes identified in previous EWASs on exposure to adverse live events^48–50^; (iii) probes identified in previous EWASs on ADHD^21,22^ or ADHD symptoms^6,9^ or (iv) probes located in ADHD-associated loci identified through GWAS^4,29,51^ showed, on average, a stronger association with adult ADHD than other methylation sites by regressing our EWAS test statistics (*Z*_score_) on each CpG category as described by van Dongen et al. ^9^:

$$|Z_{{\rm{score}}}| = {\rm{Intercept}} + \beta_{\rm{category}\,\rm{x}} *\, {\rm{category}\,\rm{x}},$$

where |*Z*_score_| represents the absolute value of the *Z*_score_ from our EWAS on adult ADHD, category x represents whether a CpG belongs or not to a specific category and $\beta$_category x_ represents the effect estimate for that category. A CpG was assigned to a category if it was associated to the phenotype of interest according to the *P*-value thresholds shown in Supplementary Table 1 [excel file]. For GWAS, we considered CpG sites within windows of 10 kb, 100 kb, and 1 Mb around significant variants (Supplementary Table 1 [excel file]). For each enrichment test, bootstrap standard errors were computed with 2,000 bootstraps using the “simpleboot” R package. Bonferroni correction was applied for multiple comparison correction (*P*_Bootstrap_ < 3.85E−03; accounting for the 13 analyses conducted).

We also tested for enrichment of regulatory domains, ontological categories and pathways, using CpG sites with *P*-value<1.00E−05 in our results. For the enrichment analysis of regulatory domains, ontological categories and pathways, probes were annotated with the Illumina Human EPIC array annotation R package (“IlluminaHumanMethylationEPICanno.ilm10b2.hg19”). The enrichment analyses for transcription factor binding sites (TFBS) and DNase I hypersensitive sites (DHS) from the ENCODE project^52^ were performed using a two-sided Fisher’s 2×2 exact test. The enrichment analyses for GO terms and KEGG, Reactome or Biocarta pathways were assessed using the *gsameth* function of the missMethyl R package^53^. Gene sets denoting canonical pathways were downloaded from MSigDB (http://www.broadinstitute.org/gsea/msigdb), which integrates Kyoto Encyclopedia of Genes and Genomes (KEGG) (http://www.genome.jp/kegg/), BioCarta (http://www.biocarta.com/), Reactome (https://reactome.org/) and Gene Ontology (GO) (http://www.geneontology.org/) resources.

The datasets for this article are not publicly available because of limitations in ethical approvals and the summary data will be available upon request.

## Results

Our sample consisted of 103 cases and 100 controls after quality control. The distribution of sexes was not significantly different between groups (χ^2^ = 2.60, *P* = 0.11), with 56% and 45% of cases and controls being male, respectively. Age of participants was significantly different between cases and controls (*P* = 3.61E−04), with a mean age of 31.90 (SD = 11.45) years in cases and of 37.25 (SD = 9.47) years in controls. In the case group, 35% of participants experienced no stressful life events, 35% were exposed to at least one prenatal stressful life event and 54% were exposed to at least one of them after birth (Supplementary Table 2; Supplementary Fig. 1).

**Fig. 1:**
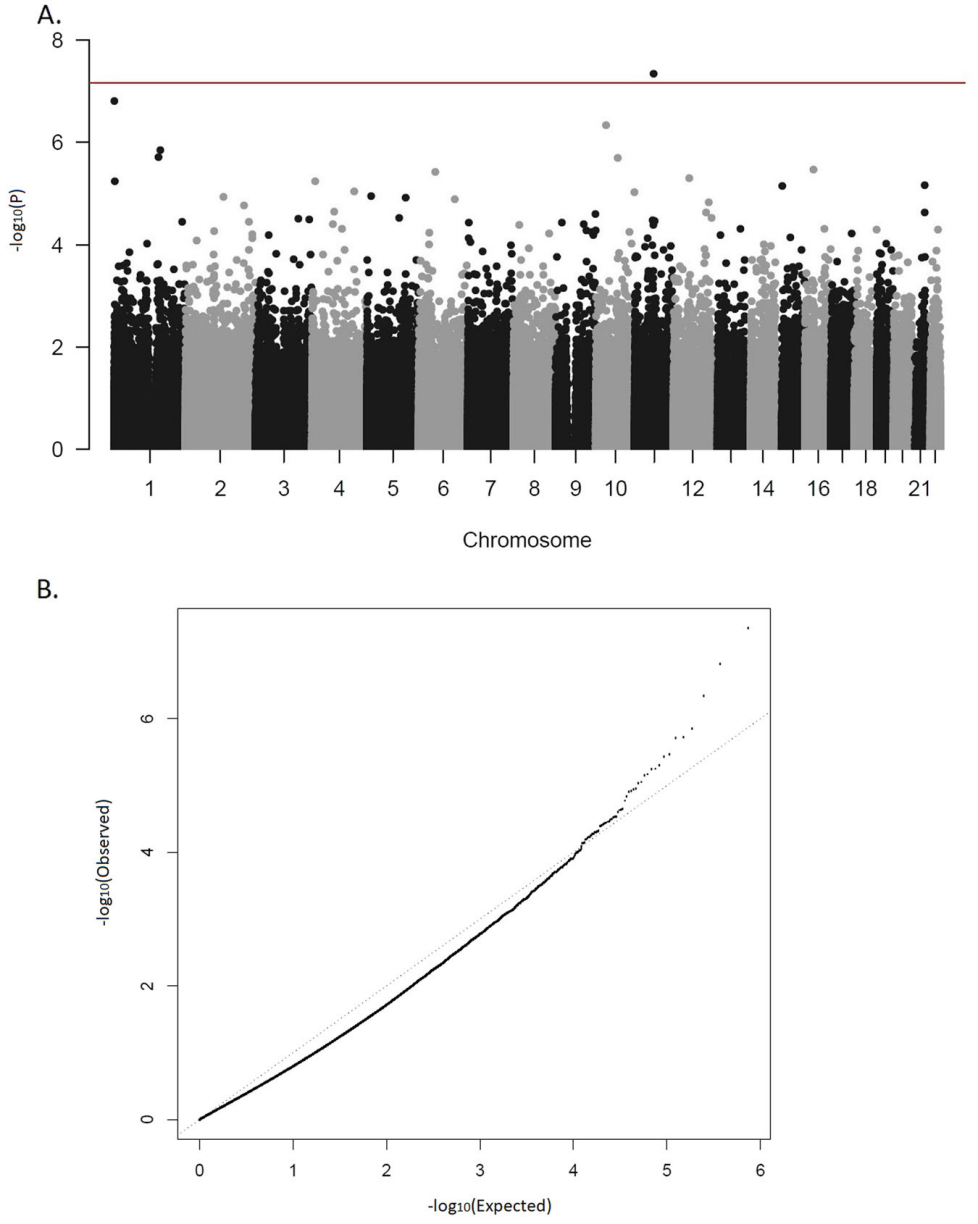
Results of the epigenome-wide association study. **a** Manhattan plot. Horizontal line indicates 5% FDR significance threshold (*P*-value=6.72E−08). **b** Quantile-quantile plot.

**Fig. 2:**
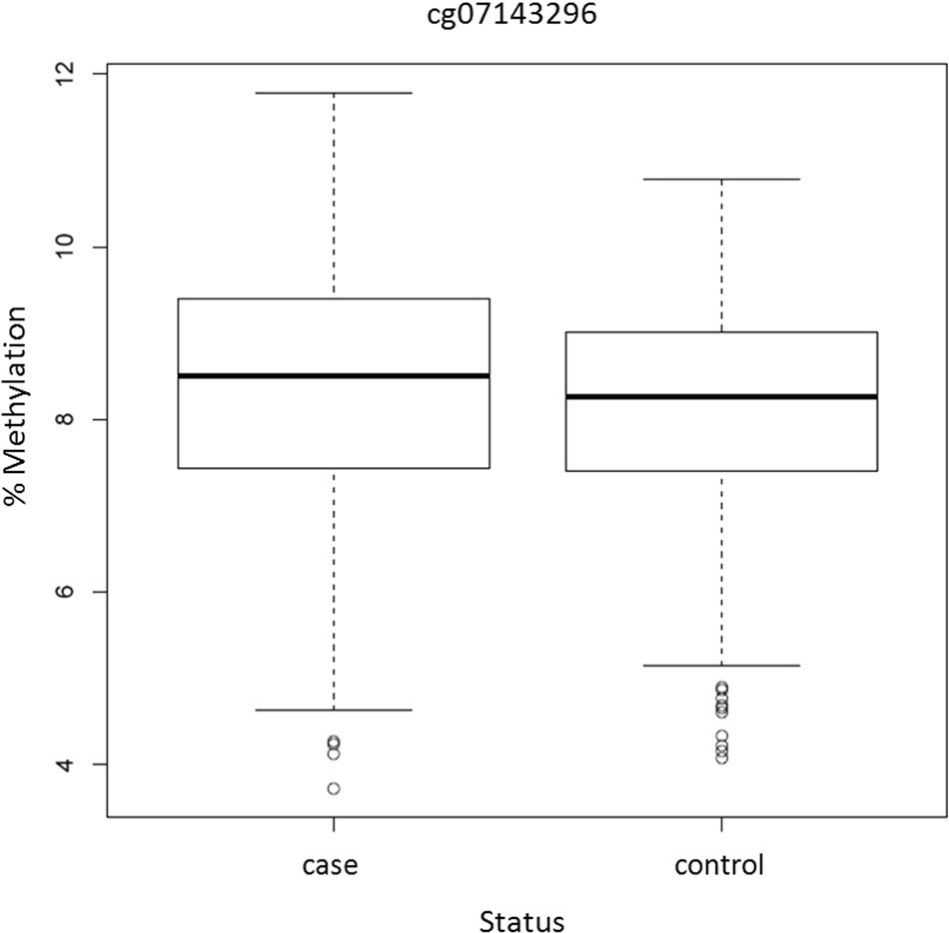
CpG‐specific DNA methylation levels. Boxplot showing the levels of DNA methylation in cases and controls at cg07143296.

We identified one differentially methylated CpG site, cg07143296, in the EWAS (P.adj = 0.033; Fig. 1a, b; Table 1; EWAS inflation factor *λ* = 0.67). This CpG lies 77 bp upstream the *PCNXL3* gene and was hypermethylated in patients, with a mean difference of 0.2% between groups (Table 1, Fig. 2). When evaluating the effect of prenatal and postnatal stressful life events on the methylation patterns of ADHD subjects at this CpG site, we found no significant differences in the methylation levels between individuals with ADHD exposed to stressful life events compared to those not exposed. The combined analysis of multiple correlated CpG sites showed evidence of association between ADHD and methylation levels in four genomic regions (P.adj < 0.02), with the most significant one spanning six CpG sites and located in the *DENND2D* gene (P.adj = 2.52E−07; Table 2). The smoking score was not significantly different between cases and controls (mean score in cases = −2.42, mean in controls = −3.34, *P* = 0.05). When we excluded it from the fitted model as a sensitivity analysis, cg07143296 (logFC = 0.0059, *P* = 1.19E−07, P.adj=0.07) and the region in chromosome 11 were no longer significant and the other regions remained significant (Table 2).

**Table 1:** Top 10 ADHD-associated differentially methylated CpG sites.

Probe ID	Location	%	logFC	P	P.adj	Gene	DHS	DHS location	Overlap transcription factor binding site
cg07143296	11: 65383707	0.24	0.0062	4.41E−08	0.033	*PCNXL3* (+77 bp)	Yes	TSS200	POLR2A | SIN3A | RBBP5 | EGR1 | YY1 | MAX | ZBTB7A | MYC
cg05041517	1: 1021029	0.24	0.0105	1.53E−07	0.057	*C1orf159*	No	–	–
cg01174734	10: 35485053	0.83	0.0152	4.54E−07	0.113	*CREM*	Yes	1st Exon; Body; 5’UTR	POLR2A | TCF7L2 | TCF12 | STAT5A | TBP | PML | NFATC1 | FOXM1 | BCL3 | EP300 | MXI1 | STAT3 | KAP1 | ATF3 | REST | GTF2F1 | JUND | MTA3 | NFIC | CEBPB | GABPA | MAX | RFX5 | TRIM28 | ATF2 | RELA | E2F1 | TAF1 | CBX3 | USF1 | RUNX3 | BCLAF1 | CREB1 | MAZ | CTCF | E2F4 | SMC3 | RAD21 | ZNF143
cg25889770	1: 161068063	0.48	0.0073	1.40E−06	0.242	*KLHDC9* (+88 bp)	Yes	TSS200	NFIC | PML | FOXM1 | SETDB1 | TFAP2C | POLR2A | RCOR1 | TBP | RFX5 | SIN3A | MXI1 | MAX | SREBP1 | HSF1 | TAF1 | SIN3AK20 | RELA | RUNX3 | YY1 | CTCF | PAX5 | POU2F2 | ZNF143 | ELF1 | BCL3 | ATF3 | SMC3
cg15705054	1: 154927709	0.36	0.0063	1.89E−06	0.242	*PBXIP1*	Yes	5’UTR	POLR2A | GATA1 | CBX3 | MAZ | MAX | SIN3AK20 | GATA2 | EP300 | SIN3A | RCOR1 | EGR1 | GABPA | TAL1 | MXI1 | RUNX3 | TCF3 | TBP | SPI1 | RFX5 | TCF12 | ELF1 | TBL1XR1 | FOXA1 | PAX5
cg05770546	10: 76004016	2.17	0.0295	1.96E−06	0.242	*ADK*	Yes	Body	CTCF | ZNF143 | RAD21 | SMC3
cg05529890	16: 28997459	0.93	0.0124	3.40E−06	0.349	*LAT*	Yes	Exon boundary; Body	–
cg23874234	6: 57087441	0.81	0.0129	3.75E−06	0.349	*RAB23* (−0.34 kb)	Yes	TSS1500	POLR2A | PHF8 | TAF1 | TCF7L2 | E2F1 | MBD4 | TBP
cg16449840	12: 54718606	0.28	0.0058	5.00E−06	0.385	*COPZ1 (+0.27kb)*	Yes	TSS1500	HDAC8 | MYC | UBTF | E2F1 | ELK1 | MBD4 | RCOR1 | FOXM1 | RXRA | CBX3 | GTF2F1 | FOSL2 | TBP | NR2F2 | JUND | BHLHE40 | IRF1 | CTCF | STAT3 | EP300 | MAFF | ZNF143 | MAX | MAZ | STAT1 | REST | GATA3 | ARID3A | SMC3 | RAD21 | RUNX3 | MAFK | MXI1 | GATA2 | USF2 | ATF3 | SIN3AK20 | USF1 | CTCFL | NFYB | GATA1 | RFX5 | E2F4 | ELK4 | ATF1
cg24479820	1: 2388073	0.91	0.0147	5.62E−06	0.385	*PLCH2* (10.83 kb)	No	–	–

Location (chromosome: base pair), DNA methylation change between groups (%), log fold change estimates (logFC), *P*-values (*P*) and adjusted *P*-values (P.adj) are shown for each CpG site.

**Table 2:** ADHD-associated differentially methylated regions identified using *comb-p*.

Chr	Coordinates	Gene annotation	CpGs	Covariates in the model	Min P	P	P.adj
1	111743038- 111743411	*DENND2D*	cg18924738, cg19269039, cg20317872, cg23184711, cg00619207, cg19268695	Sex, age, batch, smoking score and cell-type composition	8.48E−05	1.26E−10	2.52E−07
				Sex, age, batch, smoking score, cell-type composition and PRS	5.19E−06	7.25E−12	1.45E−08
	111743200- 111743411		cg18924738, cg19269039, cg20317872, cg23184711, cg00619207, cg19268695	Sex, age, batch and cell-type composition	7.73E−05	3.49E−10	1.23E−06
10	134231487- 134231549	*PWWP2B (-0.12kb)*	cg09287328, cg03860038, cg23249922	Sex, age, batch, smoking score and cell-type composition	7.61E−03	1.50E−07	1.80E−03
				Sex, age, batch, smoking score, cell-type composition and PRS	4.61E−03	8.49E−08	1.02E−03
				Sex, age, batch and cell-type composition	9.41E−04	1.52E−08	1.82E−04
11	69241075- 69241093	*LOC102724265 / AK094674*	cg22203628, cg13647725	Sex, age, batch, smoking score and cell-type composition	7.61E−03	1.18E−07	4.86E−03
				Sex, age, batch, smoking score, cell-type composition and PRS	2.28E−03	1.84E−08	7.60E−04
				Sex, age, batch and cell-type composition	1.92E−03	6.18E−08	2.55E−03
21	43823797- 43823863	*UBASH3A (* + *0.11kb)*	cg20272209, cg27280688, cg10690747, cg13578652	Sex, age, batch, smoking score and cell-type composition	1.53E−03	1.84E−06	0.02
				Sex, age, batch, smoking score, cell-type composition and PRS	4.61E−03	7.84E−06	0.09
	43823749- 43823863		cg20272209, cg27280688, cg10690747, cg13578652	Sex, age, batch and cell-type composition	2.52E−04	2.03E−09	1.33E−05

For each region, its coordinates (chromosome and start-end bp), CpGs included, minimum *P*-value in the region (Min *P*), non-corrected DMR P-value (*P*) and corrected *P*-value (P.adj) are shown along with the gene annotation obtained from the Illumina Human EPIC array annotation file and from UCSC when a gene was not annotated in a region. Results including polygenic risk score (PRS) for ADHD or excluding smoking score as covariate are also shown.

We subsequently tested whether the polygenic risk burden for ADHD had an effect on the DNA methylation signatures. After constructing PRSs at different P-value thresholds from the largest GWAS-MA on ADHD in children and adults^4^, the PRS explaining the most variance in our sample was found for *P*_T_ = 0.001 (*N*_SNPs_ = 490, *R*^2^ = 0.052, *P*_perm_ = 0.029), and was significantly higher in ADHD patients than controls (*P* = 3.10E−03; Supplementary Fig. 2). After adding it as a covariate to the model fitted for the EWAS, we found that the cg07143296 CpG site (logFC = 0.066, *P* = 1.60E−08, P.adj=0.012) and three of the four genomic regions identified remained significant (Table 2).

We then tested whether CpG sites whose methylomic variation is mainly explained by additive genetic effects showed, on average, a stronger association with adult ADHD than other methylation sites included in the array, and found a significant enrichment of signal for adult ADHD among them (*P*_Bootstrap_ = 2.39E−04). In addition, when we assessed the overlap between genetic and epigenetic signatures of ADHD, we found suggestive evidence of overlap between our EWAS results and probes annotated to ADHD-associated loci in the largest GWAS meta-analyses on ADHD across the lifespan or GWAS-MA on ADHD symptoms in children (*P*_Bootstrap_ = 6.75E−03 and *P*_Bootstrap_ = 1.36E−02, respectively), but not with results of previous GWAS-MA on ADHD conducted separately in adults or children (Supplementary Table 1 [excel file]). We also considered CpG sites differentially methylated in previous EWAS on individuals exposed to adverse life events, on clinical ADHD or on ADHD symptoms and found that CpG sites previously associated with current vs never smoking and with maternal smoking showed a highly significant enrichment of signal for adult ADHD (*P*_Bootstrap_ = 9.03E−18 and *P*_Bootstrap_ = 4.62E−14, respectively) (Supplementary Table 1 [excel file]). However, no overlap was detected with findings of previous EWASs on ADHD, on ADHD symptoms and on physical/emotional neglect or abuse (Supplementary Table 1 [excel file]).

When we focused on the top 15 differentially methylated CpG sites (*P* < 1.00E−05) in our EWAS, we found no enrichment of regulatory domains (TFBS and DHS) from the ENCODE project^52^ nor ontological categories or pathways from GO terms, KEGG, Reactome or Biocarta (Supplementary Table 3 [excel file]).

## Discussion

To the best of our knowledge, this is the first study evaluating DNA methylation signatures in a clinical sample of adults with ADHD and testing whether smoking status, polygenic risk burden for ADHD or exposure to stressful life events had an impact on the methylation signatures identified.

Methylation differences were found in regions that include genes related to cancer and pulmonary function (*DENND2D)*^54,55^, neuroticism and regulation of histone acetylation dynamics (*PWWP2B*)^56,57^ or regulation of immune signaling (*UBASH3A*)^58^. We also identified a CpG site (cg07143296) significantly hypermethylated in ADHD, located close to *PCNXL3*, a gene related to autoimmune diseases^59^. Although not achieving significance after multiple comparison correction, CpG sites in ADHD-related genes were found among the top ten signals of the EWAS, including *CREM*, which has been previously associated with impulsivity, hyperactivity, anxiety-like behavior, circadian rhythmicity and drug addiction^60–62^, *ADK*, whose deficiency may result in altered dopaminergic function, attentional impairment, and learning impairments^63,64^, or *LAT*, whose genetic variation has been associated with educational attainment^65^.

The lack of overlap between our EWAS results and those from previous EWASs on ADHD in childhood^6,10,11,20,22^ is in line with the fact that genome-wide DNA methylation is highly age dependent^34^. Contrary to some risk factors stably involved in ADHD throughout the lifespan, DNA methylation is developmental-stage specific and hence the patterns contributing to ADHD susceptibility may differ over time. The absence of overlap between our results and findings from previous EWASs on ADHD in the adulthood period^9,21^ could be ascribed to differences in the characteristics of the samples and on the array used (clinical vs population-based samples and EPIC vs Infinium Human Methylation 450K array^9^), to random variation and limited statistical power or, as previously suggested by Meijer et al.^21^, to the fact that the epigenetic effects identified may not be those with the strongest effect sizes on the phenotype^21^.

Results on the relationship between genetic and epigenetic signatures in ADHD were not conclusive. We found enrichment of signal for adult ADHD in CpGs whose methylation variance is mainly explained by additive genetic effects^47^ and suggestive evidence of enrichment in loci described in the largest GWAS-MA on ADHD^4^ and on ADHD symptoms^51^. However, no evidence was found for overlap between our EWAS results and loci from smaller GWAS-MAs on ADHD^28^ or for a substantial effect of the polygenic burden for ADHD on the methylation patterns identified. These inconsistent results should be interpreted in the context of the limited statistical power of the EWAS and warrant further investigation.

Our EWAS findings do not seem to be driven by an effect of current smoking since they were significant when we adjusted the model for it. When excluding smoking status from the model, we did not detect an effect of methylation on ADHD through smoking for cg07143296 or for the region in chromosome 11 but we cannot rule out a mediating effect for the remaining regions as their signal becomes more significant. Although bearing in mind that we used an estimated smoking score that might be a less accurate tool than clinical data, it has been postulated as a valid marker for current tobacco exposure^13,39^.

We also report preliminary data supporting overlap between epigenetic signatures of ADHD and smoking-related traits or behaviors. Enrichment of top-ranking CpGs from previous EWASs on smoking behavior^49^ or maternal smoking^50^ was obtained. In addition, methylation differences were identified in regions lying in or near genes (such as *DENND2D* or *PWWP2B*) related to phenotypes where tobacco exposure is a key risk factor^66–68^, and maternal smoking, which increases risk of ADHD in the offspring^69–71^, was the most frequently prenatal stressful life event reported by participants with ADHD.

To note, sixty-five percent of individuals with ADHD reported having been exposed to stressful life events, a circumstance that has been associated with the persistence of the disorder into adulthood^72^. Extreme familial stress was found among the most frequently reported postnatal exposures in individuals with ADHD, which is not surprising given that the presence of ADHD has been associated to varying degrees of disturbances in family and marital functioning^73–75^. However, no effect of stressful live events on DNA methylation patterns was found in ADHD subjects. Given that our study lacked data on exposure to stressful live events in controls, larger studies including cases and controls are needed to understand the impact of environmental factors on DNA methylation patterns associated with ADHD.

The results of the present study should be interpreted in the context of several limitations. First, the limited sample size of the present EWAS, which should be viewed as a pilot study whose findings await further replication. Second, our study design allowed the assessment of methylation patterns in a restricted clinical sample of medication-naïve subjects with no comorbid disorders. This design may have facilitated the identification of novel epigenetic signatures, which may not have been possible using a broader recruitment strategy. However, given that patients under medication and/or with lifetime comorbidities were excluded and this group accounts for a not negligible proportion of the overall ADHD group, further studies in larger samples including cases and controls meeting common inclusion criteria, more relaxed in terms of medication or comorbid disorders, will be required to clarify whether the results obtained could be generalized to a more realistic clinical situation. Third, the low inflation factor obtained indicates that the distribution of effect sizes in the present EWAS were not driven by systematic biases but also suggests that our study had limited statistical power and that the data may have been overcorrected, which may have prevented us from detecting methylation signatures with small effect sizes. And fourth, peripheral tissues used generally as proxies have limited utility for inferring variation in the brain^76^, although these novel signatures identified in blood might be used as biomarkers for the disorder.

In summary, we conducted the largest study assessing DNA methylation signatures in a clinical sample of adult patients with ADHD. Our results suggest that ADHD polygenic risk burden or current smoking status do not change substantially the methylomic variation between cases and controls, suggest an overlap between epigenetic signatures of ADHD and smoking-related traits, and point to an overlap between genetic and epigenetic signatures in ADHD. These results emphasize the need of additional efforts in larger samples and the inclusion of stressful life events in future studies to clarify the role of epigenetic mechanisms and environmental risk factors on ADHD across the lifespan.

## Supplementary information

Supplementary Material

Supplementary Table 1

Supplementary Table 3
