# Supplementary material for: Epigenome-wide association study of attention-deficit/hyperactivity disorder in adults
Source: Transl Psychiatry. 2020 Jun 19;10:199. doi: 10.1038/s41398-020-0860-4 (PMC7305172; doi:10.1038/s41398-020-0860-4)
Supplement: Supplementary file 1 — Supplementary Material [file 41398_2020_860_MOESM1_ESM.docx]

Supplementary Information for Rovira et al., **Epigenome-wide association study of attention-deficit/hyperactivity disorder in adults**.

**Contents**

Legends of Supplementary Tables and Figures ………………………………………………………………………………..2

Supplementary Tables……………………………………………………………………………………………………………………..3

Supplementary Figures…………………………………………………………………………………………………………………….4

**Legends of Supplementary Tables**

**Supplementary Table 1. Overlap between CpG sites identified in the present study and CpG sites or genetic variants from other studies.** [Please see Excel file]

**Supplementary Table 2. Number of stressful life events participants with ADHD have been exposed to.**

**Supplementary Table 3. Pathway analysis** [Please see excel file]

**Legends of Supplementary Figures**

**Supplementary Figure 1.** **Barplot of environmental risk factor exposure frequencies.** Prenatal and postnatal stressful life events are coloured in grey and dark grey, respectively.

**Supplementary Figure 2.** **Boxplot of polygenic risk score for ADHD in cases and controls.**

**Supplementary Tables**

**Supplementary Table 2.**

| **Number of stressful life events** |  | N individuals (%) |
| --- | --- | --- |
| During the prenatal period | 0  1  2  3  4 | 64 (65.31)  24 (24.49)  7 (7.14)  1 (1.02)  2 (2.04) |
| During the postnatal period | 0  1  2  3  4  5  6 | 45 (45.92)  21 (21.43)  16 (16.33)  7 (7.14)  3 (3.06)  5 (5.10)  1 (1.02) |
| In any period | 0  1  2  3  4  5  6  7 | 34 (34.69)  21 (21.43)  20 (20.41)  7 (7.14)  6 (6.12)  4 (4.08)  2 (2.04)  4 (4.08) |

**Supplementary Figures**

**Supplementary Figure 1.**

**
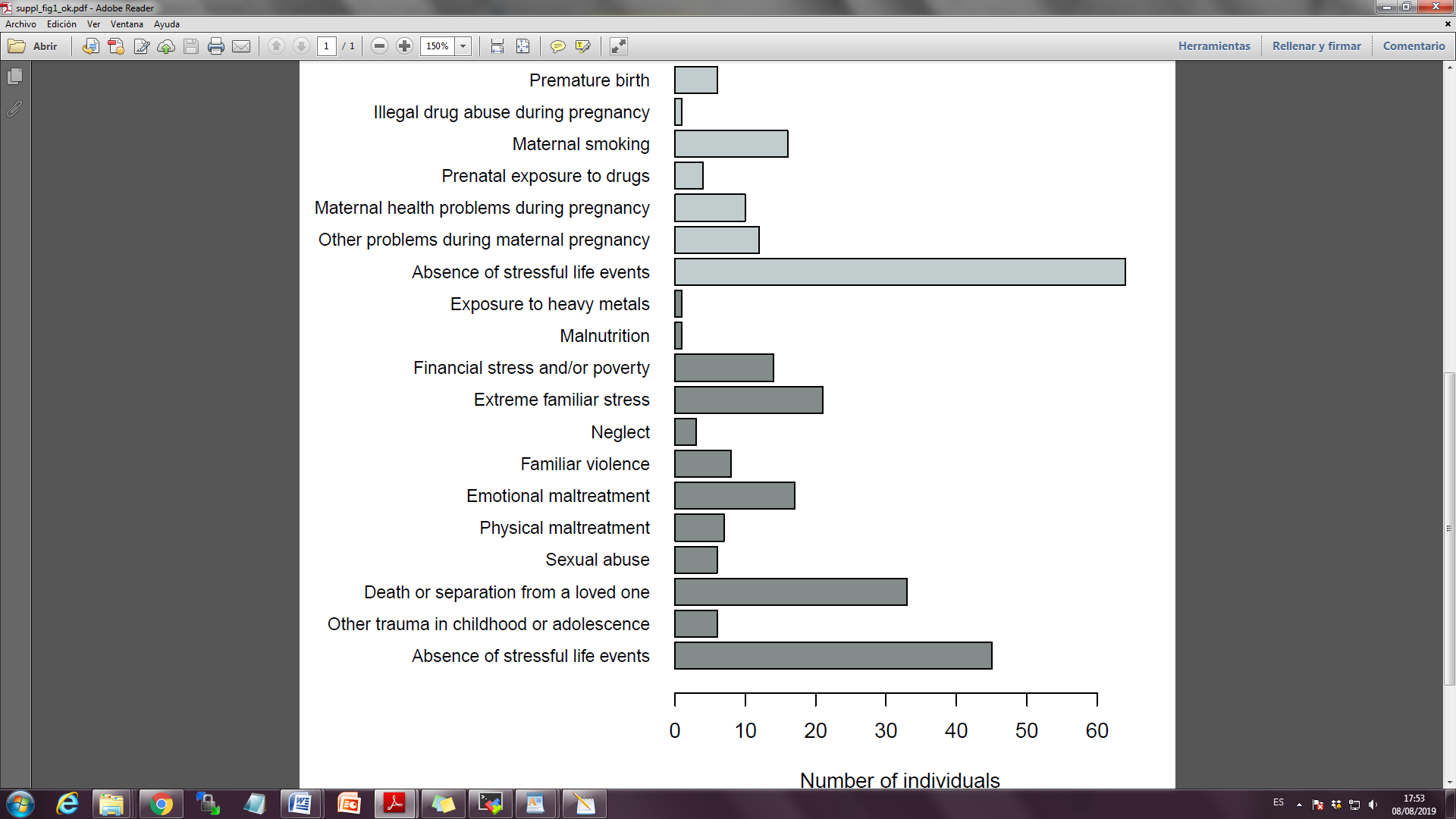
**


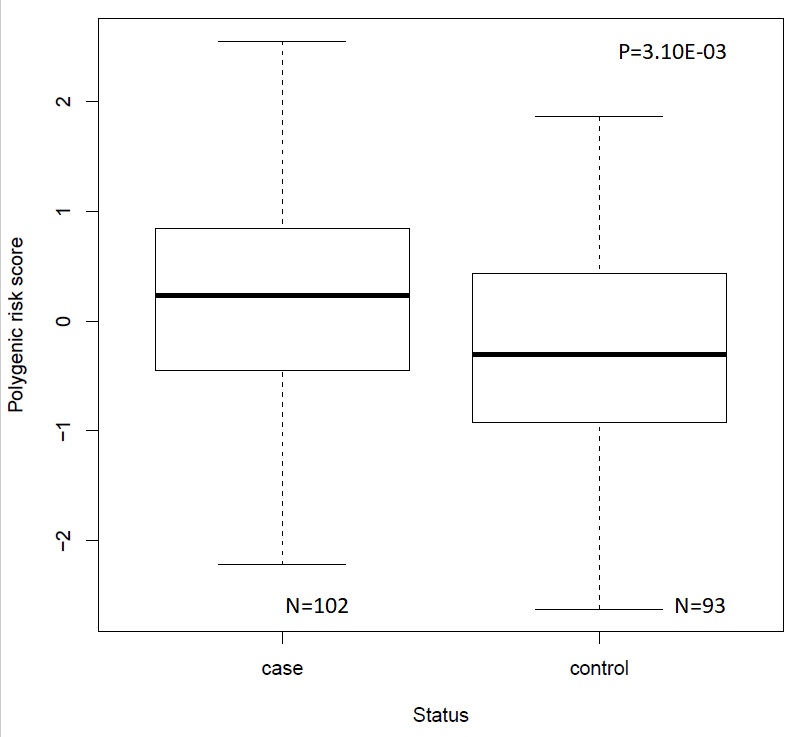
**Supplementary Figure 2.**
